# Evidence That Sri Lanka Is the Origin of a Rare Nematode Resistance Gene in Rice

**DOI:** 10.1002/pld3.70182

**Published:** 2026-07-12

**Authors:** S. Yashodha S. D. De Silva, Ceilan Handy, Eduard Roumen, M. L. A. M. S. Munasinghe, Adam H. Price

**Affiliations:** ^1^ School of Biological Sciences University of Aberdeen Aberdeen UK; ^2^ Department of Botany, Faculty of Applied Sciences University of Sri Jayewardenepura Nugegoda Sri Lanka; ^3^ Roumen Plant Breeding Support Services Herten the Netherlands

**Keywords:** evolutionary origin, landraces, *Meloidogyne graminicola*, nematode resistance genes, *Oryza sativa*, Sri Lanka

## Abstract

*Meloidogyne graminicola* is a destructive and widespread pest in tropical rice‐growing systems, whereas resistance in *Oryza sativa* remains exceptionally rare. A major quantitative trait locus (QTL) associated with resistance has previously been identified at the distal end of chromosome 11 in the resistant varieties, LD 24 (Sri Lanka) and KPM (Thailand). In this study, we combined bioinformatic and molecular approaches to characterize this locus and phenotypically screened a collection of Sri Lankan landraces to investigate the distribution and potential origin of resistance. We identified a rare 7‐bp deletion in LOC_Os11g44580 completely associated with the resistance phenotype, along with a notable occurrence of heterozygosity at this locus in some accessions. The deletion is located within a genomic region recently reported to harbor the resistance gene *MG1* in the Chinese cultivar Zhonghua 11. Among 121 Sri Lankan landraces screened using the 7‐bp deletion marker, eight accessions carried the deletion, whereas 12 were heterozygous; all exhibited resistance to Mg. Notably, the majority of resistant landraces belonged to the distinct Sri Lankan subgroup *indica 2* and were predominantly collected from the Northern Province of Sri Lanka, suggesting a potential geographic origin of this resistance‐associated allele. This study identifies 20 novel resistant varieties and highlights a regional concentration of resistance, providing valuable resources for breeding and insights into the evolutionary basis of nematode resistance in rice.

## 1 Introduction

*Meloidogyne graminicola* (Mg), a root knot nematode, is the most economically important plant‐parasitic nematode affecting rice (Bridge et al. 2005; Rusinque et al. 2021). Although primarily considered a tropical pest, it has been reported in Asia, parts of the Americas, and South Africa. More recently, it was detected in temperate rice‐growing regions of Italy in 2016 (Fanelli et al. 2017). Following this expansion, Mg was included in the European and Mediterranean Plant Protection Organization (EPPO) Alert List (“EPPO Global Database”), reflecting the increasing spread of plant pests and diseases potentially associated with climate change (Rusinque et al. 2021; Liu et al. 2023). Furthermore, future water shortages and the adoption of water‐saving practices are expected to significantly alter its geographical distribution and impact (Rusinque et al. 2021).

Mg is an obligate sedentary endoparasite that reproduces primarily through facultative meiotic parthenogenesis, although amphimixis occurs rarely (0.5%) (Mantelin et al. 2017). The first‐stage juvenile (J1) develops within the egg and molts into the infective second‐stage juvenile (J2). Upon hatching, the motile J2 either migrates through the soil or establishes a new feeding site within the same root (Kyndt et al. 2012). The J2 penetrates the root near the tip and migrates intercellularly to the zone of cell differentiation, where it induces the formation of a permanent feeding site within the stele (Gheysen and Mitchum 2011). Following establishment of the feeding site, the nematode becomes sedentary and undergoes three successive molts before reaching adulthood (Kyndt et al. 2014). Feeding activity stimulates hyperplasia and hypertrophy of surrounding plant cells, resulting in the formation of characteristic hook‐shaped galls at the root tip, a distinguishing feature of Mg (Kyndt et al. 2014). Unlike many other *Meloidogyne* species, adult females deposit eggs within host tissues, an adaptation that enhances survival under flooded conditions (Fernandez et al. 2014). The newly produced eggs give rise to J1s, which subsequently molt into infective J2s, completing the life cycle in approximately 19–27 days, depending on temperature and environmental conditions (Bridge and Page 1982). Root galling impairs the transport of water and nutrients to the shoots, leading to reduced plant vigor, significant yield losses, and aboveground symptoms including chlorosis, stunted growth, and leaf yellowing (Amarasinghe and Hemachandra 2020; Soriano et al. 2000).

Several control strategies have been employed, including the application of synthetic nematicides, continuous flooding, cultural practices such as crop rotation, and biological control agents (Amarasinghe and Hemachandra 2020). However, these approaches are often inefficient and economically unfeasible for the farmers (Mantelin et al. 2017). Consequently, natural host plant resistance is an attractive sustainable and cost‐effective alternative. For example, the resistant gene, *Mi‐1.2* from wild tomato species *Lycopersicon peruvianum* , has been commercially deployed to enhance the resistance against root knot nematodes in cultivated tomato ( *Solanum lycopersicum* ) (Milligan et al. 1998; Abad et al. 2003). Similarly, the *Ma* gene from Myrobalan plum ( *Prunus cerasifera* ) confers broad‐spectrum resistance to over 30 root knot nematode species (Lecouls et al. 1997).

To identify novel resistance genes against Mg, extensive screening of rice germplasm is required. However, natural resistance in cultivated rice is rare. Resistance has been confirmed in African rice (*Oryza glaberrima*), although introgression into Asian rice ( *Oryza sativa* ) has had limited success due to poor expression of resistance in interspecific progeny (Soriano et al. 1999; Bimpong et al. 2010). More recently, new sources of resistance have been identified in other wild rice species, such as *Oryza glumaepatula* (Mattos et al. 2019), as well as in wild rice accessions from India (Hada et al. 2020). Several studies have also reported multiple quantitative trait loci (QTL) conferring partial resistance in different mapping populations, although these require further validation. In this regard, recombinant inbred lines (RIL) populations have been widely used to map QTLs associated with Mg resistance. For example, RILs derived from a cross between Azucena and Bala ( *O. sativa* varieties) identified QTLs with partial nematode resistance on chromosomes 1, 2, 6, 7, 9, and 11 (Shrestha et al. 2007). Similarly, a cross between two *Indica* varieties Annapurna and Ramakrishna revealed two QTLs on chromosomes 1 and 3 in the RILs (Jena et al. 2013).

LD 24 (Sri Lanka) and “Khao‐Pahk‐Maw” (KPM) (Thailand) were identified as two rare resistant varieties to Mg in a genome‐wide association study of 332 accessions from a global rice diversity panel (Dimkpa et al. 2016). This study highlighted several candidate genes for further investigation, particularly those located on chromosome 11. Subsequently, Lahari et al. studied F_2_ progeny derived from crosses between the moderately susceptible *temperate japonica* cultivar Vialone Nano (VN) (Italy) and the resistant cultivars LD 24 and KPM for susceptibility to Mg (Lahari et al. 2019). Using bulk segregant analysis with the QTL‐seq approach, they identified a nematode resistance locus within the terminal 6 Mbp of chromosome 11 (23 Mbp to the end) in both crosses. More recently, Wang et al. identified a novel resistant gene, *MG1*, from the Chinese japonica cultivar Zhonghua 11 (ZH11) in the same genomic region and developed the CR24 marker for germplasm screening (Wang et al. 2023). In that study, three additional resistant varieties (SL 22‐620, HKG 98, Toga) were identified. However, Nguyen et al. reported a virulent Cambodian strain of Mg capable of overcoming the resistance of Zhonghua 11 (ZH11), KPM and LD 24 (Nguyen et al. 2023). Although this strain was unable to overcome the resistance of *Oryza glaberrima* , its emergence raises concerns about the durability of known resistant varieties and reinforces the need to identify novel sources of resistance.

In this study, we bioinformatically mined the terminal region of chromosome 11 (23 Mbp to the distal end) and identified a rare 7‐bp deletion marker at vg1126954336 in LOC_Os11g44580. We identified 20 resistant Sri Lankan landraces, of which eight carry the 7‐bp deletion and 12 are heterozygous for the marker. Notably, the majority of resistant accessions carrying the 7‐bp deletion originated from the Northern region of Sri Lanka and belong to the uniquely Sri Lankan subgroup *indica 2* (Munasinghe and Price 2016) of landraces.

## 2 Materials and Methods

### 2.1 Rice Seed Collection

A total of 135 Sri Lankan rice accessions supplied by the International Rice Research Institute (IRRI) and previously described by Munasinghe and Price (2016) were used for this study. These accessions comprised six subpopulation groups: *Tropical japonica*, *Aus*, and four *indica* subpopulations. In addition to the Sri Lankan material, two check cultivars, Azucena (*Tropical japonica*) and Bala (*indica*), were included for nematode screening using seeds from plants grown in Aberdeen that were originally obtained from IRRI. Detailed information on all accessions is available in the IRG Seed Request List of accessions. As the seed collection was 12 years old, some accessions failed to germinate. In total, 121 accessions were genotyped (using the 7‐bp marker), whereas 65 accessions were phenotyped in resistant screening experiments.

### 2.2 Bioinformatic Analysis of Region 23‐Mb end of Chromosome 11

Genome sequences of KPM, LD24, and Vialone Nano (VN) generated by Lahari et al. were used in the bioinformatic analysis (Lahari et al. 2019). All annotated resistance genes within the target region (23 Mb to the distal end of chromosome 11) were identified and evaluated using the Rice Genome Project (RGP) database (http://rice.uga.edu/index.shtml) (Kawahara et al. 2013). Candidate genes were selected based on the following criteria: identical sequences between the resistant varieties (KPM and LD24) (100% homology); sequence divergence (< 100% homology); from the susceptible cultivar VN and the reference genome Nipponbare (which is susceptible) and the presence of exceptionally rare polymorphisms (a frequency in the region of 1% or less based on the study of Dimkpa et al. (2016) (Wang et al. 2023)). The presence and structure of each candidate gene were examined using Integrative Genomics Viewer (IGV). Sequences were visually compared to assess overall similarity and shared allelic variation between LD24 and KPM that differed from VN and the reference sequence of Nipponbare. Shared SNPs unique to KPM and LD24 that differed from VN and Nipponbare were recorded for future analysis. Genes were excluded if they were absent, identical to VN and Nipponbare, or showed nonallelic variation between KPM and LD24, as these genes are unlikely to underlie shared resistance. It is important to note that certain genes contained regions with problematic alignments, characterized by high levels of apparent heterozygosity, likely resulting from the misalignment of reads from paralogous genes to a single reference locus.

#### 2.2.1 RiceVarMap SNP Acquisition and Filtering

A list of variant loci (SNPs and INDELs) was obtained by Rice Variation Map (RiceVarMap2) (Zhao et al. 2015). Genes confirmed to be present and similar between KPM and LD24 were submitted to the “Variation by Gene” function in RiceVarMap, with upstream and downstream regions set to 0 bp prior to analysis. For haplotype analysis, up to 100 SNPs were selected. INDELS and synonymous SNPs were excluded, although rare INDELs of potential interest were noted for subsequent investigation. When fewer than 100 SNPs remained after filtering, all available SNPs were used. For LOC_Os11g44580 where more than 100 SNPs were retained, random sampling was performed to select 100 SNPs for analysis. During randomization, SNPs were ordered numerically and assigned either 1 or 2. SNPs assigned the value 1 were retained for haplotype analysis.

#### 2.2.2 RiceVarMap haplotype network analysis

Filtered SNP data were submitted to the “Classification 2” analysis in RiceVarMap, which groups accessions into the following subpopulations: *Indica*, *Aus*, *Aromatic*, *Tropical japonica*, and *Temperate japonica*, using the “Haplotype Network Analysis” function. As haplotype sequences for KPM, LD24, and VN were not available in RiceVarMap, corresponding haplotypes were manually reconstructed using base pair information from IGV in combination with variant ID data. The reconstructed sequences were then compared with haplotype groups generated by RiceVarMap to identify identical or closely related haplotypes.

#### 2.2.3 Identification of Accessions Potentially Containing Resistant Allele

SNPs and INDELs unique to both LD24 and KPM were examined using RiceVarMap's “Variation information by Variation ID” function to assess their distribution across *O. sativa* subpopulations. SNPs/INDELs that were exceptionally rare, shared between LD24 and KPM, and absent from both VN and the Nipponbare reference genome were considered putative diagnostic markers for resistance. Accessions carrying these candidate resistance‐associated polymorphisms were identified, and available genotypes were subsequently grown and phenotyped for resistance to Mg.

### 2.3 Molecular Screening

Genomic DNA was extracted using Qiagen DNeasy Plant Mini Kit (Qiagen, Germany). PCR amplification was performed using Thermo Scientific Arktik Thermal Cycler (Cat. No. N11467, Finland) under the following conditions: initial denaturation at 95°C for 5 min; 35 cycles of 94°C for 30 s, 58°C for 30 s, and 72°C for 1 min; followed by a final extension at 72°C for 5 min and a 4°C hold. For amplification of the 7‐bp deletion marker at vg1126954336 in LOC_Os11g44580, primers flanking the target region were used (forward: GTGCCTCCGAATACTTCACG; reverse: GGCGAACATCTCCAGGAAGA). PCR products were separated on 4% high resolution agarose gel at 400 V for 80 min using a BIO‐RAD gel electrophoresis system. Similar PCR protocols were used to screen the “T” SNP at vg1126379478 in LOC_Os11g43700 and the CR24 marker described by Wang et al. (2023). For amplification of the region containing the “T” SNP, the following primers were used; forward primer—CCCTTCTTCTCCAGCAACAAC; reverse primer—CGACTGCAAAGCTTGTTTCTG. The PCR products were digested with VspI (AseI) and resolved on 2% agarose gels at 400 V for 40 min. For screening with the CR24 marker, primers TCGTCATGTGGTTTCTGGC (forward) and TGTTGCAGTTGCTTCTGC (reverse) (Wang et al. 2023) were used, and PCR products were analyzed on 2% agarose gels under the same electrophoresis conditions.

### 2.4 Nematode Culture and Inoculation

Mg was provided by the Ghent University and maintained on *O. sativa* cv. Azucena. Nematode screening experiments were conducted in the greenhouse at the University of Aberdeen under controlled conditions of 26°C/24°C day/night temperature, under an 11 ½‐h‐day/night light regime and about 70% relative humidity. A modified version of the protocol described by Shrestha et al. (2007) was used for nematode extraction, in which infected root galls were macerated and incubated in petri plates containing distilled water for 1 week prior to inoculation. Three independent screening runs were conducted to evaluate a total of 65 accessions, with some repeated due to low germination. In each run, 28 accessions were screened alongside two check varieties, Azucena (*Tropical japonica*) and Bala (*indica*) using four 6 × 9 plug trays. Each tray contained seven accessions plus the two check varieties, with six replicate plants per accession. Rice seeds were sown in sand‐filled plug trays (two seeds per well), and seedlings were thinned to one plant per well 1 week after sowing. Plants were watered daily and fertilized twice weekly with Yoshida's solution (Yoshida et al. 1976). Fourteen‐day‐old seedlings were inoculated with 250 J2 per plant and gall numbers were recorded 14 days postinoculation (dpi).

#### 2.4.1 Resistance Screening of Vellai

Vellai and Azucena (check) were sown in a 6 × 9 plug tray in alternating rows, with a total of 27 replicates per accession. The screening protocol described above was followed. Each Vellai plant was genotyped for the 7‐bp deletion marker and the T SNP marker.

### 2.5 Investigating the Dominant Effect of the Allele

A progeny population of 52 plants derived from selfing a heterozygous individual of Sinnavellai (IRGC 11946) was used for nematode screening. A comparable number of Azucena plants were included as the check variety. Progeny plants were sowed in alternating rows with Azucena in a randomized arrangement. All plants were genotyped using the 7 bp deletion marker. Mean gall counts were compared among plants having the heterozygous and homozygous nondeletion genotypes. Statistical analyses were performed using one‐way analysis of variance (ANOVA), followed by Turkey's pairwise comparison and a chi‐square test at a 5% significance level.

### 2.6 Resistant Screening With Nematode Populations From Sri Lanka

Accessions with low gall counts that carried the 7‐bp deletion were screened using a nematode population from Sri Lankan. The same screening protocol described above was followed.

### 2.7 Statistical Analysis

To account for variation between trays or screening runs, the mean gall numbers of replicate plants for each accession were divided by the combined mean of the check genotypes Azucena and Bala within the corresponding tray or screening run. Significant differences among genotypes were evaluated using one‐way ANOVA, followed by Tukey's post hoc test at a 5% significance level. A chi‐square goodness‐of‐fit test was also performed.

## 3 Results

### 3.1 Bioinformatic Analysis of Candidate Genes at the End of Chr 11 (23Mbp Downstream)

According to Lahari et al. (2019), the terminal 6‐Mbp region of chromosome 11, spanning from 23 Mbp to the distal end, was identified as a target region harboring a nematode resistance QTL. This region contained a total of 861 genes, of which 606 genes remained after excluding the 255 transposons and retrotransposon‐related genes. Among these, 29 were annotated as “hypothetical proteins” and 231 as “expressed proteins.” A subset of 47 genes was annotated as resistance‐related, including seven classified as “disease resistance” genes, 11 containing “NB‐ARC” domains, and 16 belonging to the NBS‐LRR class, along with others annotated as “RGH,” “rust resistance,” or “stripe rust resistant” proteins. All resistance‐related genes within this region were subjected to bioinformatic screening (Table S1). Sequence data from LD 24 and KPM were analyzed using IGV, and candidate genes were selected based on the following criteria: identical sequences between LD 24 and KPM; divergence from the susceptible cultivar Vialone Nano and the reference genome Nipponbare; and the presence of rare polymorphisms, consistent with the rarity of resistance. The filtering process is summarized in Table S2. Of the 47 genes analyzed, four were absent in both KPM and LD24, three absent in KPM, and two were absent in LD24. For 20 genes, KPM and LD24 did not share the same allele, including seven cases where high heterozygosity in sequence alignments prevented reliable allele resolution. A further 16 genes were excluded due to extensive heterozygosity in both genotypes, suggesting unreliable alignment. Following this filtering process, only LOC_Os11g43700 and LOC_Os11g44580 remained as potential candidate genes for Mg resistance. LOC_Os11g43700 was annotated as a *RGH1A gene*, while LOC_Os11g44580, annotated as an NBS‐LRR gene, was prioritized as the top candidate. Notably, LOC_Os11g44580 showed high similarity to the *Aegilops tauschii* gene *Go35* and *Cre3‐*, *CreZ*‐, and *Cre8v* resistance genes, which are known to confer resistance to *Heterodera avenae* in wheat (de Majnik et al. 2003).

**FIGURE 1:**
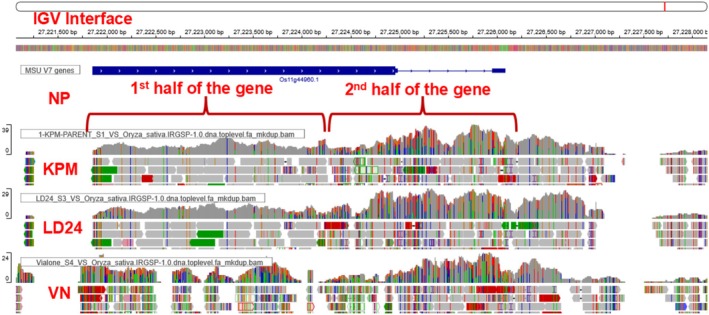
IGV view of sequence alignment of LD24, KPM, and Vialone Nano (VN) with reference sequence of Nipponbare (NP) in the gene LOC_Os11g44960.

Wang et al. identified a 52.2‐kb region of Nipponbare containing four annotated NLR—like genes (LOC_Os11g44960, LOC_Os11g44970, LOC_Os11g44990, and LOC_Os11g45050) in the region corresponding to the *MG1* gene in ZH11 (Wang et al. 2023). However, these genes were not included among the candidate genes proposed by Lahari et al. as they did not meet the selection criteria applied in that study (Lahari et al. 2019). Closer inspection of sequence alignments revealed that the initial portion of LOC_Os11g44960 shows similarity between LD24 and KPM but differs from both Nipponbare and Vialone Nano. In contrast, the latter portion of this gene exhibits substantial apparent heterozygosity (Figure 1), making it difficult to resolve its precise sequence structure in LD24 and KPM. This pattern may reflect misalignment arising from the presence of closely related paralogous sequences in this genomic region. The remaining annotated genes in this region (LOC_Os11g44970, LOC_Os11g44990, and LOC_Os11g45050) were not considered further, as there is no evidence supporting their presence in LD24 and KPM; specifically, no sequence reads from these accessions aligned to the corresponding regions of the Nipponbare.

### 3.2 Haplotype Analysis in Determining the Rarity of Alleles in Candidate Genes

**FIGURE 2:**
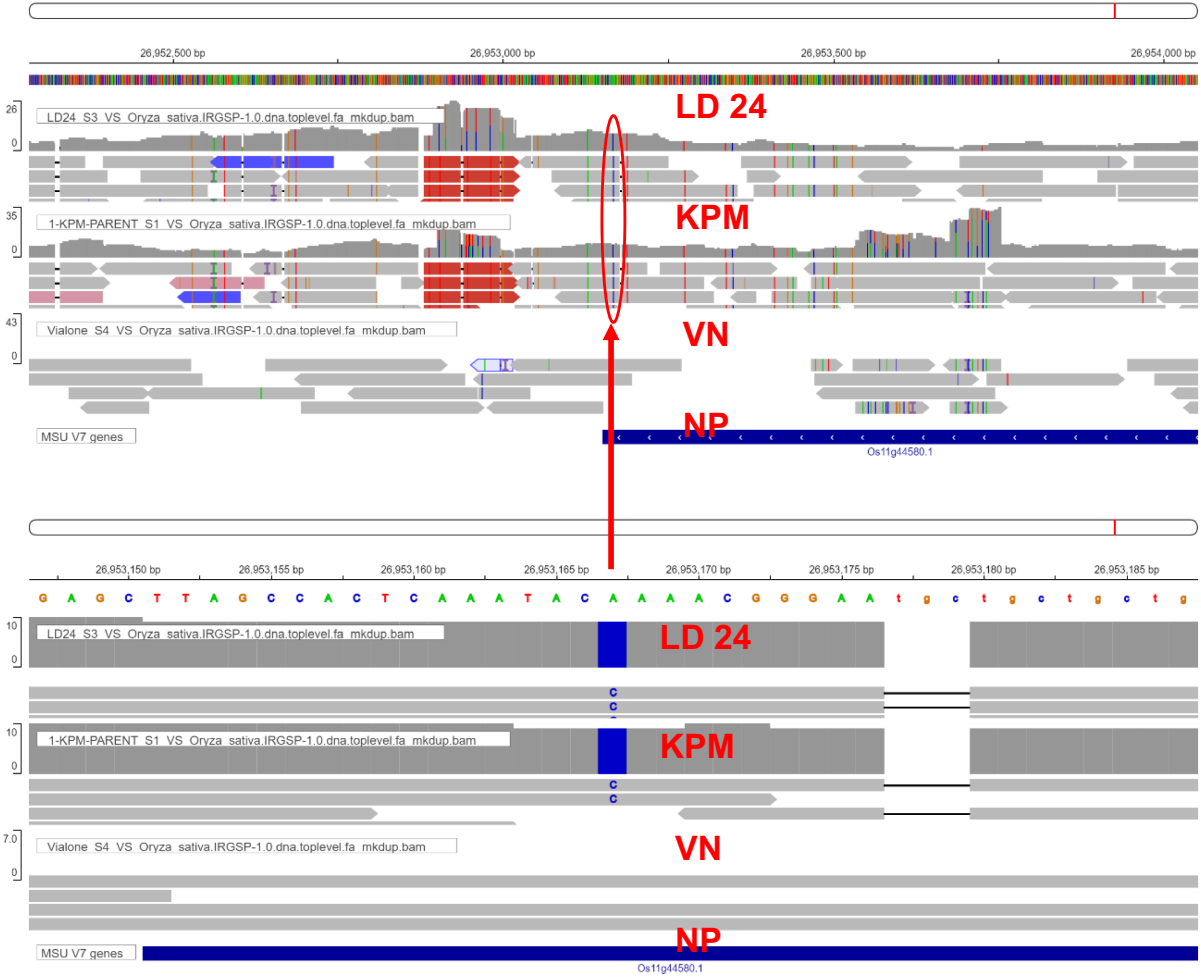
The “C” SNP identified at vg1126953167 in LD 24 and KPM (resistant varieties) but absent in Vialone Nano (susceptible Italian variety) viewed under IGV.

Two complementary approaches were used to identify rare SNPs and indels. First, sequence data from LD 24, KPM, and Vialone Nano were visualized through IGV to detect polymorphisms shared between the resistant accessions (LD 24 and KPM) but absent in susceptible accessions (Vialone Nano and Nipponbare), and vice versa (Figure 2). Second, RiceVarMap (Zhao et al. 2015) was used to analyze the rare polymorphisms and to identify the haplotype groups within the two most promising candidate genes LOC_Os11g43700 and LOC_Os11g44580.

**FIGURE 3:**
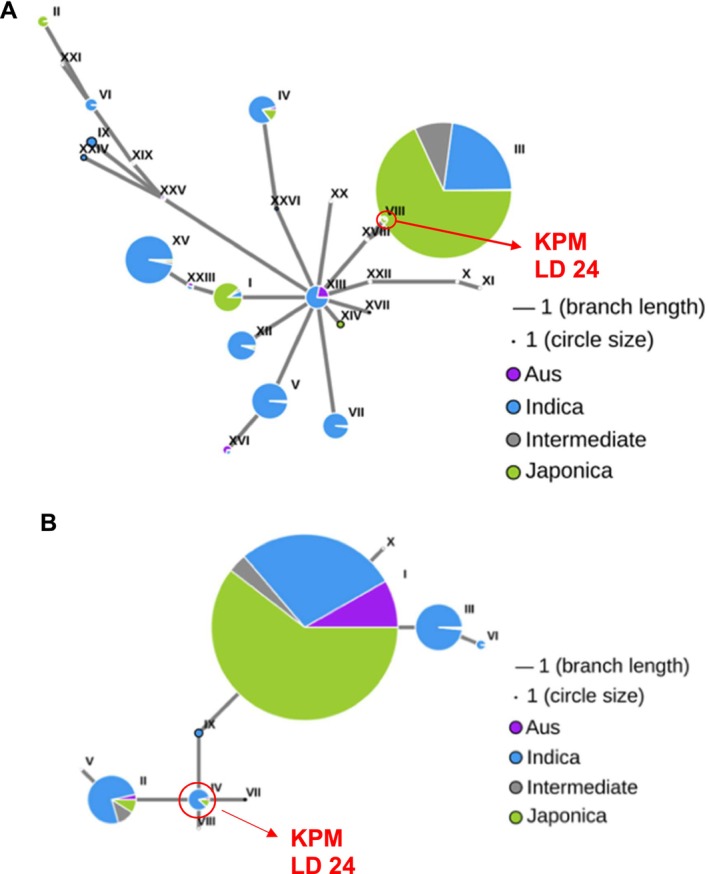
Haplotype plots of two candidate genes: (A) 26 haplotype groups of LOC_Os11g43700 derived from 73 SNPs entries in RiceVarMap (Zhao et al. 2015). KPM and LD24 haplotype sequences are identical to the relatively small group VIII, mainly consisting of the *Japonica* subpopulation. (B) Ten haplotype groups of LOC_Os11g44580 derived from 72 SNP entries in RiceVarMap. KPM and LD24 are nearly identical to group IV. This near identical group consists of mostly of the *Indica* subpopulation, with some *Japonica* present.

LOC_Os11g43700 spans 26,376,803–26,380,866 bp and contains 153 variant positions. Haplotype analysis based on 73 nonsynonymous SNPs identified 26 distinct haplotype groups (Figure 3A). As LD 24, KPM, and VN are not accessions represented in the RiceVarMap (Zhao et al. 2015) database, sequence polymorphisms for these accessions were examined using IGV to determine their corresponding haplotypes. Both LD24 and KPM were assigned to haplotype group “VIII,” which comprises 65 accessions, the majority of which belonged to the *Japonica* subpopulation (Figure 3A). Variant IDs associated with haplotype group “VIII” were further analyzed to identify rare SNPs. Among these, a “T” SNP at vg1126379478 was identified as a rare variant and considered a potential candidate associated with Mg resistance. This SNP has an allele frequency of 1.40% and is predominantly distributed among *Japonica* and *Aus* accessions. Notably, most of the 65 accessions within haplotype group “VIII” carrying this “T” allele originate from the Philippines, Sri Lanka, and China.

### 3.3 Preliminary Genotype and Phenotype Screening on Similar Named Cultivars Identified in the Haplotype Analysis for Resistance

During haplotype analysis, both genes LOC_Os11g43700 and LOC_Os11g44580 exhibited rare polymorphisms that appeared potentially diagnostic of resistance. To further evaluate the association between these polymorphisms and resistance to Mg, selected cultivars carrying these variants were subjected to phenotypic screening. Initially, three accessions with available sequence data in the RiceVarMap2 database were chosen for testing: Heendikwee (IRIS_313‐107020), Moddai Karuppan (IRIS_313‐9862), and Podiwee1 (IRIS_313‐9831). These cultivars were consistently identified among the accessions carrying the candidate diagnostic polymorphisms (Table 1) and originated from Sri Lanka.

**TABLE 1:** RiceVarMap (Zhao et al. 2015) output for vg1126954336, vg1126953167, and vg1126379478 allele variation of in potential resistant cultivars.

Variation ID	vg1126954336 (7‐bp indel)	vg1126953167 (A‐>C)	vg1126379478 (C‐>T)
Variation type	INDEL	SNP	SNP
Reference	GCCATTCT	A	C
Primary allele	GCCATTCT	A	C
Secondary allele	ACCATTCT	C	T
IRIS_313‐10720 (HEENDIKWEE)	GCCATTCT	C	T
IRIS_313‐9831 (PODIWEE)	G	C	C
IRIS_313‐9862 (MODDAI_KARUPPAN)	G	C	T

**FIGURE 4:**
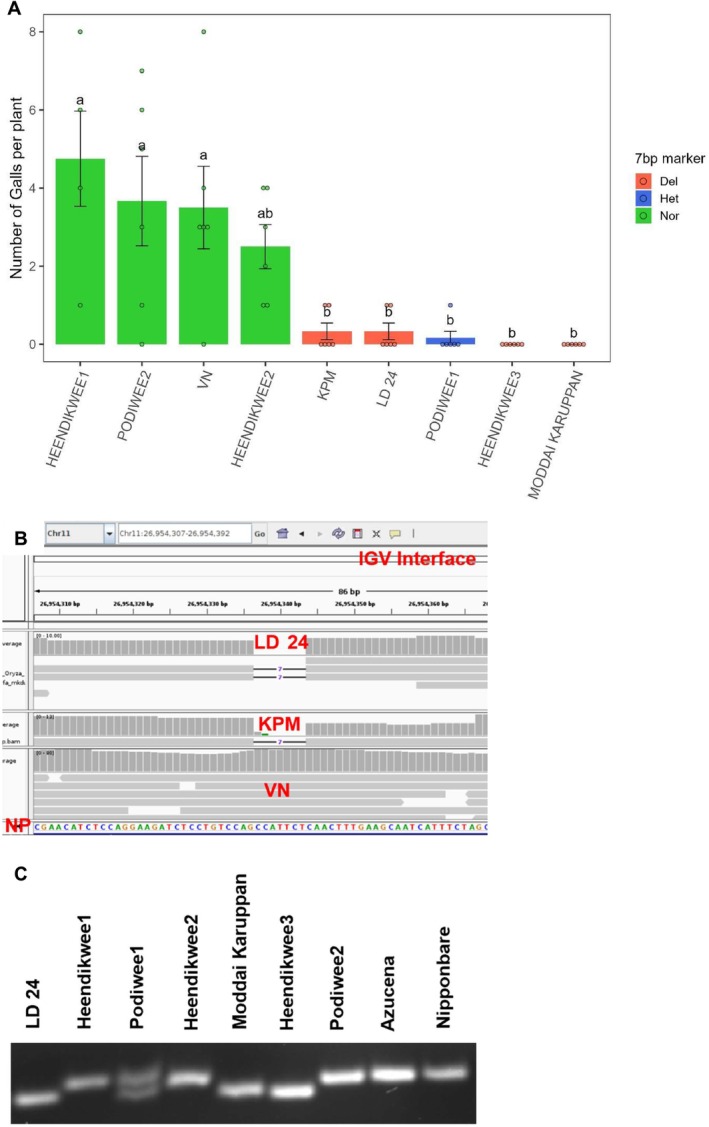
(A) Number of galls on six Sri Lankan landraces, positive control (resistant—KPM and LD24), negative control (susceptible—Vialone Nano VN) at 14 dpi. Data are means ± SEM (*n* = 6 independent plants). Different letters above the bars indicate statistical significance groups at *p* < 0.05 (one‐way ANOVA analysis followed by Tukey post hoc test). (B) 7‐bp deletion (at vg1126954336) identified in LD 24 and KPM (resistant varieties) but absent in Vialone Nano (susceptible Italian variety) viewed under IGV. (C) Molecular screen of six Sri Lankan accessions (Heendikwee1, Heendikwee2, Heendikwee3, Moddai Karuppan, Podiwee1, and Podiwee2) using a 7‐bp deletion marker at vg1126954336 in LOC_Os11g44580. LD 24 was used as the resistant check, whereas Azucena (AZ) and Nipponbare (NP) were used as the susceptible checks (Original gel image provided in Figure S1).

Gall counts were recorded 2 weeks after inoculation with Mg. At this time point, approximately one life cycle of the nematode is expected to have been completed, which may account for the relatively low gall numbers observed even in susceptible cultivars (Figure 4A). Infection was observed in the majority of tested cultivars. The known resistant cultivars KPM and LD 24, and the susceptible VN, exhibited the expected infection patterns. Resistant cultivars (KPM and LD24) showed fewer than 0.33 galls on average, whereas susceptible cultivars exhibited more than 3 galls (Figure 4A). A one‐way ANOVA (*F* = 7.358, *R* = 57.8%, *p* < 0.001) followed by Tukey's HSD test revealed significant differences among cultivars. KPM, LD24, Podiwee1, Heendikwee3, and Moddai Karuppan formed a statistically distinct resistant group with significantly lower gall counts compared to Vialone Nano, Podiwee2, and Heendikwee1, which constituted a susceptible group. Heendikwee2 did not differ significantly from either group (Figure 4A). Based on their gall counts relative to resistant controls, Heendikwee3, Moddai Karuppan, and Podiwee1 were identified as putatively resistant cultivars.

The 7‐bp deletion at vg1126954336 in LOC_Os11g44580 (Figure 4B) was used as a molecular marker to initially screen these 6 Sri Lankan accessions (Heendikwee1, Heendikwee2, Heendikwee3, Moddai Karuppan, Podiwee1, and Podiwee2). LD 24 was included as the resistant control, whereas Azucena (AZ) and Nipponbare (NP) were used as susceptible controls. Consistent with phenotypic data, LD 24, Heendikwee3, Moddai Karuppan, and Podiwee1, identified as resistant based on low gall counts, displayed a lower PCR band corresponding to the presence of the deletion. In contrast, Heendikwee1, Heendikwee2, Azucena (Az), and Nipponbare (Np) showed an upper band, indicating the absence of the deletion (Figure 4C). Interestingly, Podiwee1 exhibited two bands, suggesting heterozygosity at this locus. It is important to note that DNA used for molecular screening was pooled from the six replicate plants per accession. Therefore, the observed banding patterns may reflect within‐accession variation, indicating the possible presence of both resistant and susceptible individuals within the same accession.

### 3.4 Testing Sri Lankan Cultivars for Rare Alleles Associated With Resistance

Following this preliminary molecular screen, 121 Sri Lankan cultivars (initially obtained from IRRI for the study of Munasinghe and Price 2016) were further analyzed using this 7‐bp marker. In addition, a subset of 15 accessions (Table S3) was screened for the T SNP at vg1126379478 (C‐>T) in LOC_Os11g43700 as well as for the CR 24 marker associated with the *MG1* locus described by Wang et al. (2023). Among the 121 accessions screened using the 7 bp marker, eight were identified as homozygous for the deletion, whereas 12 accessions were heterozygous, including the previously characterized accessions Heendikwee3, Moddai Karuppan, and Podiwee1.

Among 15 accessions screened for the T SNP marker (Table S3), Kirinaran, Periya Sivappu, and Vellai showed discrepancies among the three markers (T SNP marker and 7 bp marker and CR 24 marker). In Kirinaran, the T allele was absent, whereas the accession was heterogeneous for both the 7 bp and CR 24 markers. Periya Sivappu was heterozygous for the T SNP marker but showed a homozygous deletion for the 7 bp and CR24 markers. In contrast, Vellai was homozygous for the T SNP marker but heterozygous for both the 7 bp and CR24 markers. Initially molecular screening was performed using pooled DNA samples. To further resolve these inconsistencies, individual plant‐level genotyping was conducted on 16 plants of Vellai. Both the 7‐bp deletion and CR24 showed polymorphism among individual plants, whereas the T marker was present in all individuals (Table S4). Of the 16 plants analyzed, nine carried the 7‐bp deletion and the corresponding CR24 band indicative of resistance. No heterozygous individuals were detected for the T SNP marker. Notably five plants exhibited a discrepancy between genotype and phenotype, displaying high gall numbers (Kyndt et al. 2014; Fernandez et al. 2014; Bridge and Page 1982; Amarasinghe and Hemachandra 2020; Soriano et al. 2000; Milligan et al. 1998; Abad et al. 2003; Lecouls et al. 1997; Soriano et al. 1999; Bimpong et al. 2010; Mattos et al. 2019; Hada et al. 2020; Shrestha et al. 2007; Jena et al. 2013; Dimkpa et al. 2016; Lahari et al. 2019; Wang et al. 2023) despite carrying the T allele. Statistical analysis revealed a strong association between the 7 bp and CR24 marker genotypes and gall count (*F* = 20.4, *p* < 0.001), whereas the association with the T SNP marker was weak (*F* = 4.9, *p* = 0.043). Therefore LOC_Os11g43700 was excluded as a potential candidate based on lack of a perfect association between the markers (T SNP, 7 bp, CR 24) and phenotyping results in Kirinaran, Periya Sivappu and Vellai.

### 3.5 Phenotypic Screening of Landraces to Identify Associations Between the Markers and Resistance

**FIGURE 5:**
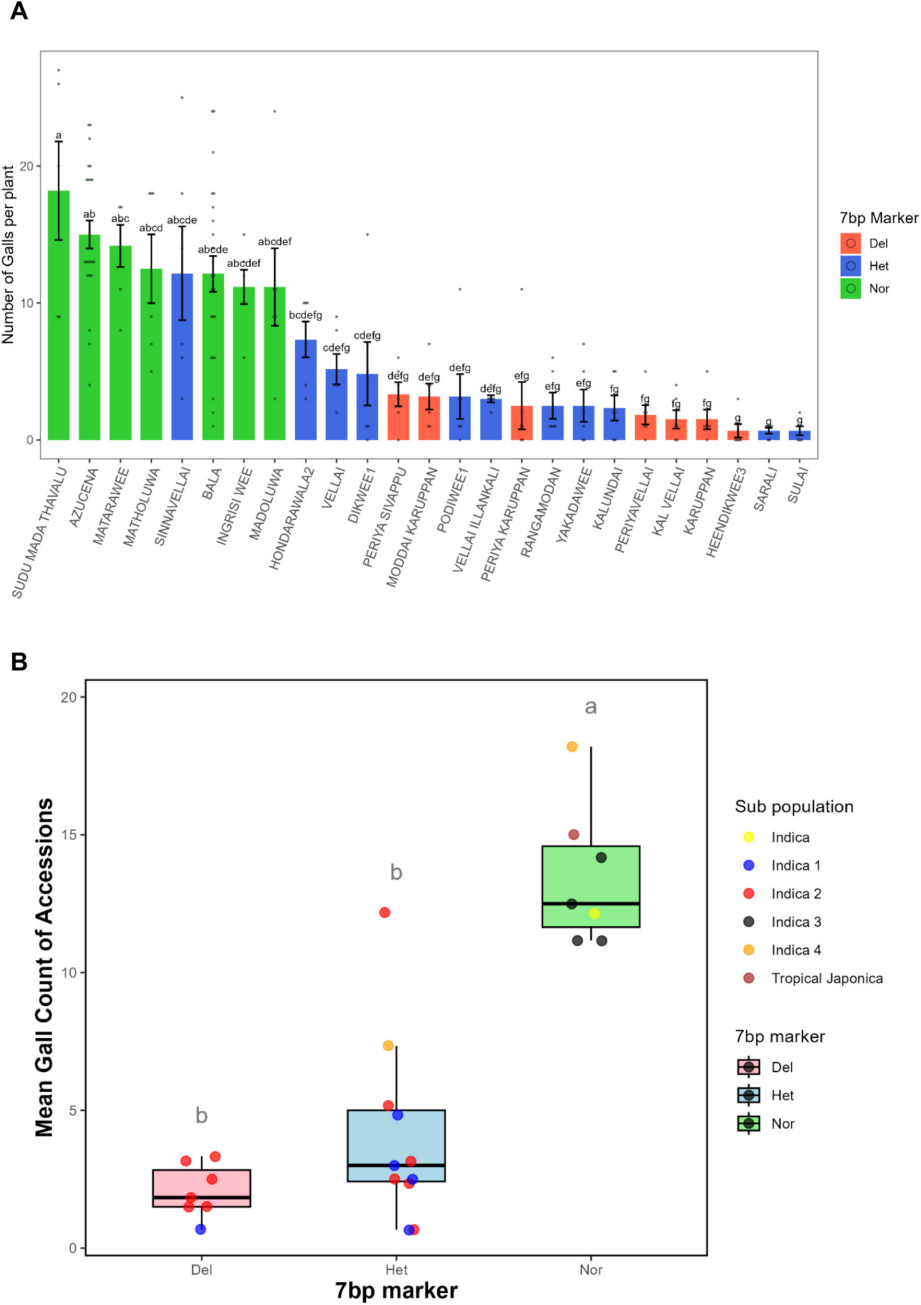
(A) Number of galls per plant of Sri Lankan landraces at 14‐dpi screened in single screening run color coded by 7‐bp marker. Data are means ± SEM (*n* = 4–6 independent plants). Different letters above the bars in indicate statistical significance groups at *p* < 0.05 (one‐way ANOVA analysis followed by Tukey post hoc test). The molecular screening was done pooling DNA from all 4–6 individual replicates of each genotype. Del and Nor denotes genotypes with and without the 7‐bp marker, respectively. Het denotes the genotypes having individual plants within an accession with and without the 7‐bp marker. (B) Box plot of the distribution of mean gall counts of accessions screened in a single screening run grouped by the 7‐bp marker. Individual data points color coded by the *Oryza sativa* subpopulation identified in Munasinghe and Price (2016). Check varieties Azucena and Bala used in the screen belong to the subpopulations *Tropical japonica* and *Indica* (separate from the subpopulations of Munasinghe and Price (2016)). Different letters above the bars indicate statistical significance groups at *p* < 0.05 (one‐way ANOVA analysis followed by Tukey post hoc test).

Phenotyping was subsequently conducted for 65 accessions, the majority of which were pre‐screened by the 7‐bp marker. Seeds were randomly selected from batches provided by IRRI to minimize sampling bias. As shown in Figure 5A, mean gall counts at 14 dpi from a single screening experiment are presented and color coded according to 7‐bp deletion marker genotype, with accessions arranged in descending order of mean gall count. Landraces carrying the 7‐bp deletion consistently exhibited the lowest mean gall counts, whereas those lacking the deletion showed the highest gall counts. A one‐way ANOVA revealed a highly significant difference in mean gall counts between genotypic classes (*F* = 38.36, *p* = 6.73 × 10^−8^, *R* ^2^ = 0.78). In particular, landraces carrying 7‐bp deletion (including heterozygotes) differed significantly from those lacking the deletion (denoted as Nor in Figure 5B), which exhibited substantially higher gall counts. These results demonstrate a strong association between the 7‐bp deletion marker and nematode resistance as measured by gall formation.

### 3.6 Exploring the Nature of Heterozygosity

**FIGURE 6:**
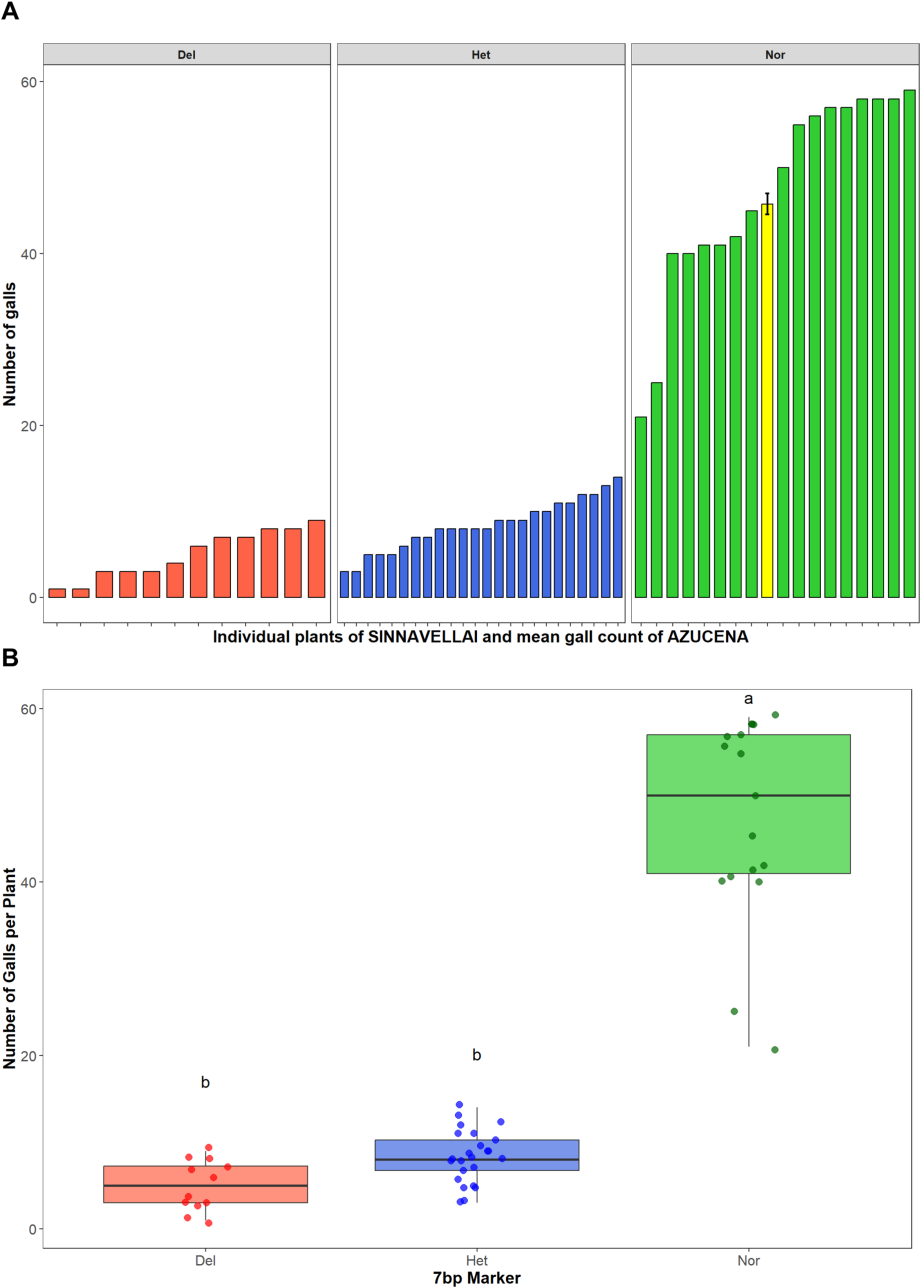
(A) Gall numbers of individual plants of Sinnavellai (a progeny of a heterozygous parent) in a single screening run with Azucena (check) plants grouped by the presence of the 7‐bp marker (the mean gall number is given with the standard error bar in a single bar in yellow). (B) Box plot of the same distribution Figure 6. (A) of gall numbers of individual plants of Sinnavellai grouped by the 7‐bp marker. Different letters above the bars indicate statistical significance groups at *p* < 0.05 (one‐way ANOVA analysis followed by Tukey post hoc test).

Heterozygosity for the 7‐bp marker was initially observed in 12 accessions including Vellai, Sinnavellai, Kalundai, Podiwee1, Yakadawee, Sulai, Kirinaran, Dikwee1, Rangamodan, Sarali, Vellaiillankali, and Hondarawala2, based on genotyping of pooled DNA samples derived from multiple plants per accession. To validate these observations at the individual plant level, we genotyped replicate plants from selected accessions (Vellai, Sinnavellai, Kalundai, and Podiwee1) using the 7‐bp marker. Among these, true heterozygous individuals were confirmed only in Sinnavellai. Although heterozygosity appeared relatively frequent at the accession level (detected in pooled samples from four accessions), it was rare at the individual plant level, as demonstrated by its detection in only a single accession (Sinnavellai, IRGC 11946). To further investigate the inheritance pattern and potential dominance of the resistance‐associated allele, we generated a progeny population of 53 plants derived from a single heterozygous individual of Sinnavellai (IRGC 11946). Each progeny plant was phenotyped for nematode resistance and genotyped using the 7 bp marker. As shown in Figure 6, gall numbers are presented for individual plants, color‐coded according to marker genotype. We identified 12 plants carrying the 7‐bp deletion (putative resistant homozygotes), 24 heterozygous plants, and 17 plants lacking the deletion (putative susceptible homozygotes). A one‐way ANOVA revealed a highly significant effect of genotype on gall numbers (*F* = 184.9, *p* < 2 × 10^−16^). Post hoc comparisons using Tukey's HSD test indicated that plants lacking the deletion exhibited significantly higher gall numbers than both deletion‐carrying and heterozygous plants, whereas no significant difference was observed between the latter two groups. Furthermore, a chi‐square goodness‐of‐fit test showed that the observed segregation ratio of resistant to susceptible plants (36:17) did not significantly deviate from the expected Mendelian 3:1 ratio (*χ* ^2^(1) = 1.42, *p* = 0.23). Together, these results are consistent with resistance being controlled by a single dominant allele associated with the 7‐bp deletion marker.

### 3.7 Exploring the Geography and Genetic Populations of Origin of Resistance

**FIGURE 7:**
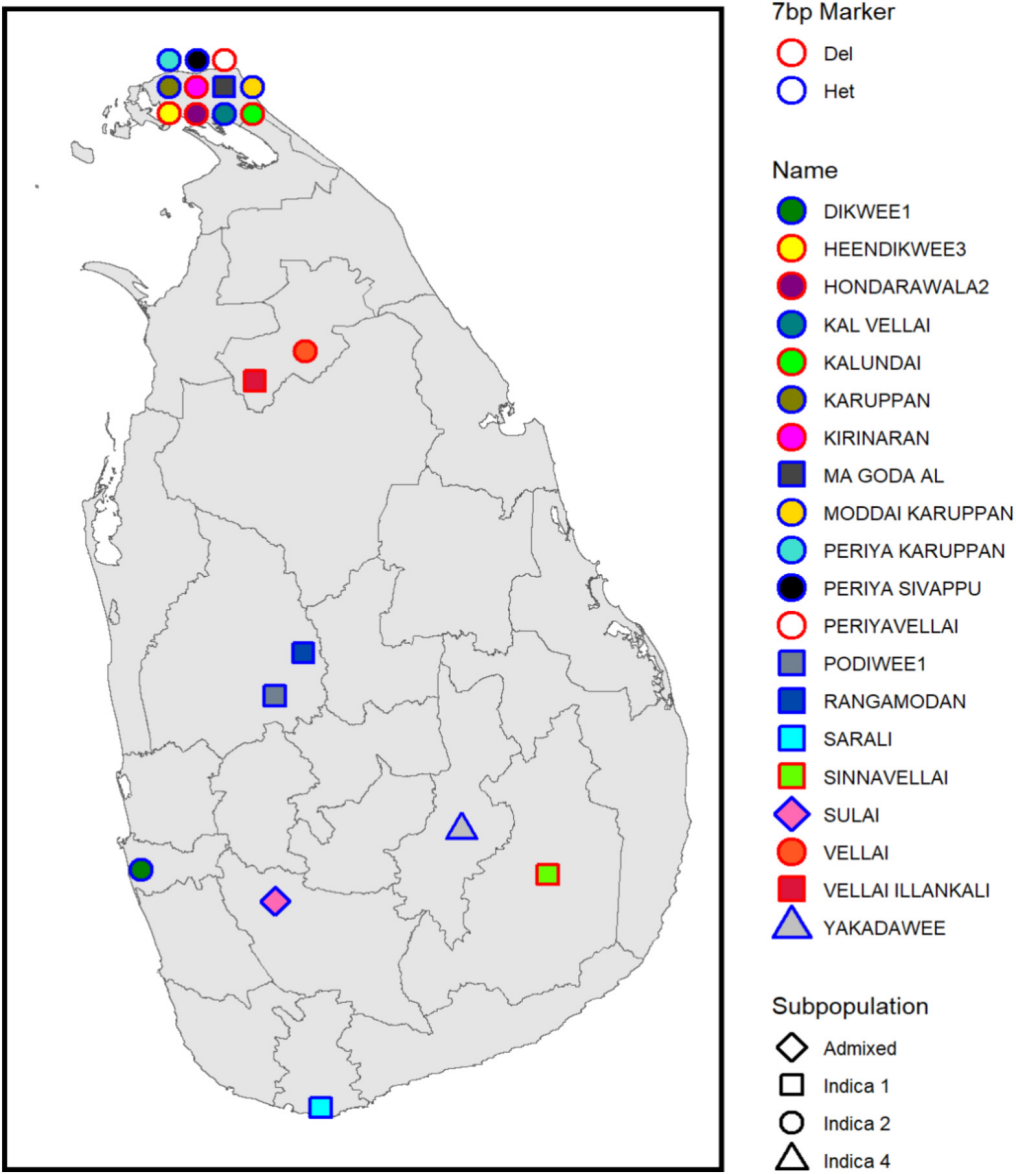
Distribution of Sri Lankan landraces having the 7‐bp deletion (either homozygous or heterozygous) grouped by the subpopulation (identified in Munasinghe and Price 2016).

**FIGURE 8:**
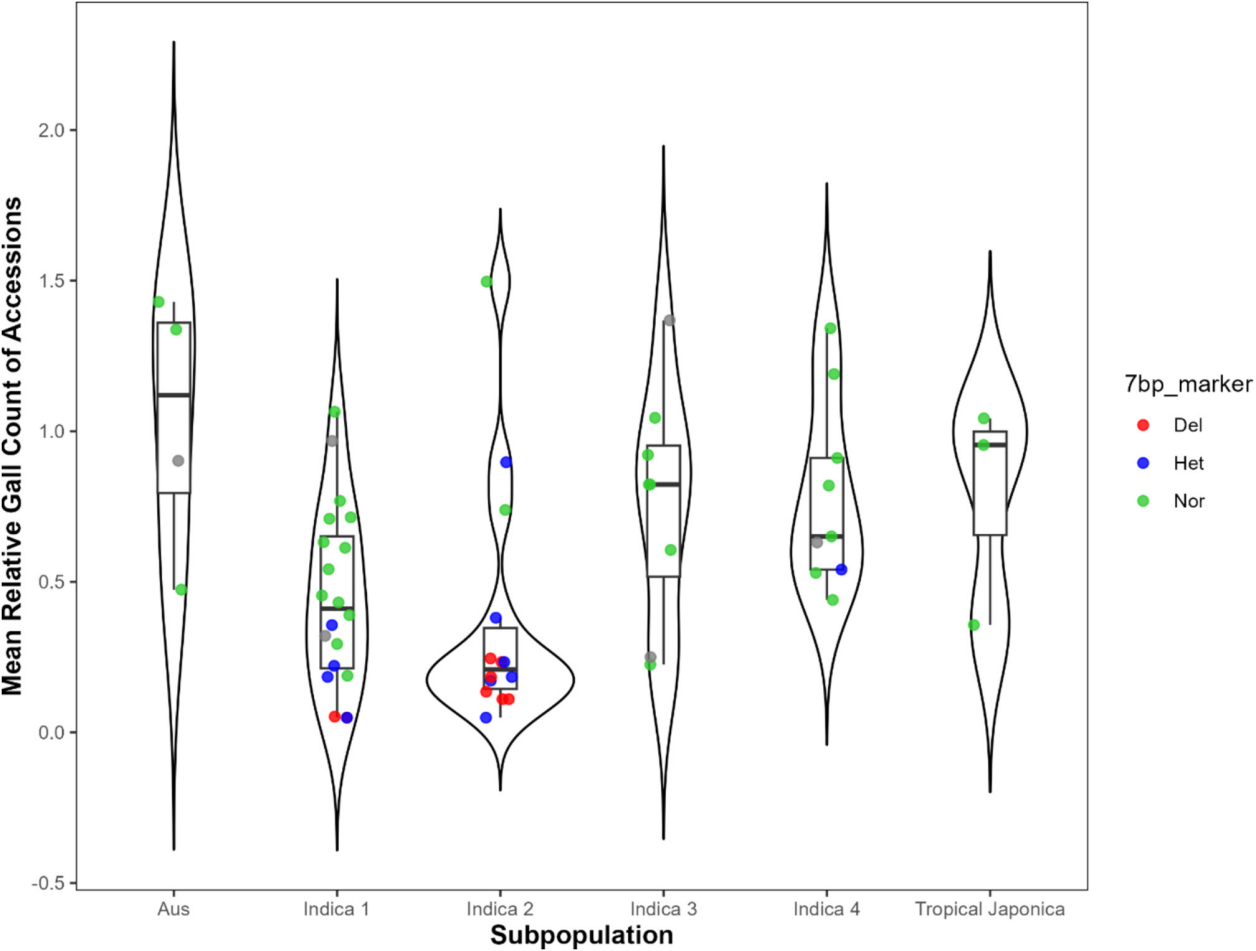
Violin plot of mean relative gall counts of landraces belonging to unique Sri Lankan subpopulations described in Munasinghe and Price 2016 grouped by the 7‐bp marker (Data points are colored in gray for few accessions where molecular data are absent).

To investigate the geographic distribution and genetic population structure underlying resistance, we mapped the landraces carrying the 7‐bp deletion including the heterozygotes. As shown in Figure 7, landraces classified as “Del” and “Het” exhibit a nonrandom distribution, being predominantly concentrated in the Northern Province and largely belonging to the uniquely Sri Lankan *indica* 2 subpopulation described by Munasinghe and Price (2016). We further examined the relationship between the 7‐bp marker and phenotypic resistance by analyzing the mean relative gall counts of 65 landraces from the Sri Lankan subpopulations defined by Munasinghe and Price (2016). As illustrated in the violin plot in Figure 8, the majority of the landraces carrying the 7‐bp deletion (including heterozygotes) display low mean relative gall counts and belong to the uniquely Sri Lankan *indica* 2 subpopulation described by Munasinghe and Price (2016).

To better understand the genetic structure and potential evolutionary dynamics of resistance, we analyzed allele frequencies among the 121 landraces genotyped for the 7‐bp deletion (Table 2), assuming a dominant resistance allele (R). Since each landrace carries two alleles, the total allele count in the population was 242, comprising 28 resistant (R) alleles (from RR and Rr genotypes) and 214 susceptible (r) alleles (from Rr and rr genotypes). When allele frequencies were compared across provinces, a distinct pattern emerged: the Northern Province differed markedly from other regions, showing a higher frequency of the R allele (62.5% compared to 11.6% across the country) and a lower frequency of the susceptible homozygous (rr) genotype (37.5% compared to 88.4% nationwide). This geographic enrichment further supports the nonrandom distribution of the resistance‐associated marker and suggests possible localized selection or population structure effects.

**TABLE 2:** Allele frequencies within 121 landraces in each province of Sri Lanka.

Province	Total landraces	RR_resistant	Rr_heterozygous resistant	rr_suseptible	R_resistant allele frequency%	rr_suseptible allele frequency%
Central	7	0	0	7	0	100
Eastern	3	0	0	3	0	100
North Central	5	0	0	5	0	100
North Western	12	0	2	10	8.3	91.7
Northern	16	7	6	3	62.5	37.5
Sabaragamuwa	17	0	1	16	2.9	97.1
Southern	36	0	1	35	1.4	98.6
Uva	12	1	1	10	12.5	87.5
Western	9	0	1	8	5.6	94.4

## 4 Discussion

### 4.1 Introduction

*Meloidogyne graminicola* (Mg) is widely recognized as one of the most economically important nematode pests affecting rice, due to the substantial yield losses it can cause under favorable environmental conditions. In this context, breeding strategies must anticipate future challenges associated with Mg spread and prioritize the development of resistant rice varieties. In this study, we identified 20 resistant Sri Lankan landraces and a “7‐bp deletion” diagnostic marker associated with resistance phenotypes. Among these, 11 landraces were heterozygous and segregated for resistance, producing both resistant and susceptible individuals in self‐fertilized progenies. Notably, the majority of the resistant accessions belonged to the uniquely Sri Lankan *indica 2* subgroup described by Munasinghe and Price (2016) and were predominantly derived from the Northern Province of Sri Lanka. Although similar findings of varying degrees of susceptibility have been reported in other studies (Soriano et al. 1999; Shrestha et al. 2007; Plowright et al. 1999), this study claims a higher number of resistant *O. sativa* accessions, the majority of which originate from a singular locality of a country while sharing the same resistant allele. It is important to note that, at the time this research was conducted, structural variation within the target genomic region (such as gene duplications or large insertions/deletions) could not be comprehensively assessed. As such, additional genomic complexity linked to the resistance phenotype may not have been fully captured in this study. Further studies, including knockout phenotyping, protein functional analyses, and mechanistic investigations, will be required to establish causality.

### 4.2 Is Sri Lanka the Origin of a Rare Nematode‐Resistant Gene?

Despite advances in genotyping technologies, the exploration and characterization of local genetic diversity in Sri Lankan rice remain limited. Even in major genetic diversity panels and sequencing projects such as the Rice Diversity Panel and the 3 K Rice Genome Project, Sri Lankan rice is underrepresented. The study by Munasinghe and Price highlighted the distinct genetic diversity present in Sri Lankan landraces (Munasinghe and Price 2016), identifying the *Indica 2* subpopulation as a unique subgroup specific to this region with germplasm and genetic variation that remain poorly characterized in global collections. Our findings further support the presence of unique genetic attributes within Sri Lankan landraces, particularly with respect to nematode resistance. The observed enrichment of resistance‐associated alleles within this subgroup raises the possibility that Sri Lanka may represent a previously underexplored reservoir of rare resistance genes, warranting further investigation into its evolutionary and functional significance.

*Myrmecina graminicola* was first reported in 1997 in Sri Lanka and has since been recognized as widespread across rice‐growing regions of the country (Rusinque et al. 2021). However, it is likely that the nematode was present long before its initial documentation, potentially exerting sustained selection pressure on local rice populations. Given that wild relatives typically harbor greater genetic diversity than domesticated cultivars, it is plausible that variation for nematode resistance may have originated from these taxa under prolonged evolutionary pressure. In particular, wild rice species growing in environments with high nematode prevalence would be expected to evolve resistance mechanisms. Consistent with this, a recent study reported that the majority of accessions resistant to Mg belonged to *Oryza nivara* (Hada et al. 2020). Although these accessions were collected from the middle Gangetic plains agroclimatic region, *O. nivara* is also present in Sri Lanka and may represent a similar reservoir of resistance alleles. This wild relative is often used to enhance genetic diversity by the IRRI (Nair 2019), the institute where this study sourced its known resistant cultivars. Based on these observations, we hypothesize that resistance alleles identified in this study may have originated from *O. nivara* populations in the dry zone low‐country regions of Northern Sri Lanka and subsequently introgressed into local landraces. The geographic concentration of resistant accessions in these regions is consistent with this hypothesis. Furthermore, early human migration and settlement patterns, particularly those originating from the Indian subcontinent into Northern Sri Lanka may have facilitated the dissemination of such resistant germplasm across the country.

Allele frequency patterns provide important insights into the genetic structure, evolutionary dynamics, and selective pressures acting on a population. The predominance of the susceptible *rr* genotype (83.5% of landraces) suggests several possible explanations. First, resistance may not have been under strong positive selection, potentially due to historically low nematode pressure. Second, the resistance allele (*R*) may have been introduced relatively recently into the gene pool, possibly through gene flow from wild relatives. Third, the homozygous resistant genotype (*RR*) may have been selected against due to potential fitness or yield costs. Consequently, farmer‐mediated selection practices may have prioritized yield over resistance, thereby constraining the expansion of the *R* allele. This could explain the continued predominance of the susceptible genotype (*rr*) and suggests that the selective advantage conferred by resistance may not have been sufficient to drive fixation of the allele within the landrace population.

### 4.3 Heterozygosity in Resistant Accessions

Although the precise cause of the observed heterozygosity remains unclear, we propose several plausible explanations. Initial genotyping based on pooled DNA samples of multiple plants per accession suggested the presence of heterozygosity in several landraces. However, when individual plants from selected accessions were genotyped, true heterozygosity was rarely detected. Instead, the apparent heterozygosity at the accession level likely reflects seed mixtures, where seed batches supplied by IRRI contained a combination of individuals carrying different alleles rather than truly heterozygous plants. This observation suggests that the original collected material may have been heterogeneous, comprising both allelic types and that subsequent seed multiplication and maintenance through repeated cycles of selfing and propagation led to the fixation of alleles within individual plants, resulting in predominantly homozygous genotypes. An alternative explanation for the observed heterozygosity is gene duplication, whereby some accessions may carry multiple copies of the gene, including both deletion and nondeletion variants. However, the scarcity of heterozygous individuals at the single‐plant level, together with the Mendelian 1:2:1 segregation pattern observed in the progeny of a heterozygous Sinnavelai plant (Figure 6) argues against this possibility.

Given that true heterozygosity was rarely detected, we nonetheless considered whether the *Rr* genotype might confer an evolutionary advantage. Heterozygosity could enhance adaptive flexibility by maintaining both resistant (*R*) and susceptible (*r*) alleles. In dynamic environments where nematode virulence shifts, (Rr) individuals might retain partial resistance, whereas (rr) individuals remain fully susceptible. Additionally, certain resistance mechanisms may function more effectively in a heterozygous state, enabling a broader defense response. Although this hypothesis can be tested by evaluating (*RR*), (*Rr*), and (*rr*) genotypes against geographically diverse nematode populations, our current data provide limited evidence that heterozygote advantage plays a major role in maintaining variation at this locus.

### 4.4 Is the 7‐bp Deletion Allelic With *MG1*?

In a recent study Wang et al. (2023) described *MG1* as a resistant gene that is highly expressed at sites of nematode infection in several rice varieties, including ZH11, LD 24, and KPM. The authors further reported that *MG1* resides within a 75.4‐kb genomic region containing eight expressed genes, which is absent in the reference genome Nipponbare, and replaces the deleted 52.2‐kb region encompassing genes LOC_Os11g44960‐45050. Notably, *MG1* is located within the same genomic locus as our candidate gene (LOC_Os11g44580). LOC_Os11g44580 is positioned approximately 250‐kb upstream of *MG1*. The markers associated with these two loci (7‐bp deletion and CR24 marker) are therefore in close proximity, suggesting that they may be genetically linked and unlikely to segregate independently over a limited number of generations. However, the distance between them is sufficient that recombination events could, in principle, separate the two loci. If no accessions show discordance between the two markers, this would provide strong evidence of linkage. Conversely, the presence of multiple accessions exhibiting contrasting genotypes between the markers would indicate recombination and argue against tight linkage. Thus, examining the cosegregation patterns of these markers across diverse germplasm provides a useful approach to assess their genetic relationship.

It is important to note that the deleted 52.2‐kb region of the Nipponbare genome, which contains four annotated NLR‐like genes (LOC_Os11g44960, LOC_Os11g44970, LOC_Os11g44990, and LOC_Os11g45050), shows poor alignment when compared with the corresponding sequences from LD24 and KPM. This could be due to sequencing errors. Although this discrepancy could reflect technical factors such as sequencing or assembly errors, it may also indicate underlying structural variation between the resistant LD24/KPM genotypes and the susceptible Nipponbare reference. Such variation could include gene presence–absence differences, duplications, or larger insertions/deletions that are not accurately captured in the current reference‐based alignment. To resolve this ambiguity, targeted de novo sequencing and assembly of this genomic region in LD24 and KPM will be necessary to accurately characterize its structure and gene content.

Another area of uncertainty concerns the distribution and potential introgression of the resistance‐associated allele across diverse rice subpopulations and geographic regions. For example, resistant accessions examined in this context include LD 24 (an *indica* variety from Sri Lanka), KPM (an *Aus* variety from Thailand), ZH11 (a *temperate japonica* variety from China), SL 22‐620 (an *Aus* variety from Sierra Leone), and HKG 98 (an *Aus* variety from Mali). The presence of resistance‐associated alleles across such diverse genetic backgrounds and geographic origins raises questions about their evolutionary history and dissemination. One plausible explanation is that resistance alleles originated in wild rice populations and were subsequently introgressed into cultivated rice through hybridization and gene flow. Over extended timescales, processes such as natural selection, early agricultural practices, and human‐mediated seed exchange and migration may have facilitated their spread across geographically and genetically distinct populations. Although this scenario is plausible, it remains to be empirically validated. To better understand these evolutionary dynamics, comprehensive sequence comparisons across diverse rice germplasm will be essential to elucidate the origin, diversity, and distribution of resistance‐associated alleles. Furthermore, given the ongoing evolutionary pressure imposed by pathogen virulence, strategies such as gene pyramiding and the deployment of diverse resistance sources will be critical for enhancing the durability of nematode resistance. Finally, further investigation of the *MG1* locus may provide valuable insights into the origin and stability of resistance, particularly in the context of known variation in pest virulence.

## Author Contributions

**S.Y.S.D.D.S., A.H.P.:** conceptualization. **S.Y.S.D.D.S., A.H.P.:** methodology. **S.Y.S.D.D.S.:** formal analysis (data analysis and statistics). **S.Y.S.D.D.S., C.H.:** experimental work. **S.Y.S.D.D.S., A.H.P., E.R.:** bioinformatics. **A.H.P., M.L.A.M.S.M.:** seed acquisition. **S.Y.S.D.D.S.:** data curation. **S.Y.S.D.D.S.:** writing – original draft. **S.Y.S.D.D.S., A.H..P., M.L.A.M.S.M., C.H., E.R.:** review and editing. **S.Y.S.D.D.S.:** visualization. **A.H.P., M.L.A.M.S.M.:** supervision. **S.Y.S.D.D.S.:** funding acquisition. All authors read and approved the final manuscript.

## Funding

S. Yashodha S. D. De Silva was supported by a Split PhD Commonwealth Scholarship (ref no. LKCN‐2021‐364).

## Conflicts of Interest

The authors declare no conflicts of interest.

## Supporting information


**Data S1:** Peer review.

**Figure S1:** Molecular screen of six Sri Lankan accessions (Heendikwee1, Heendikwee2, Heendikwee3, Moddai Karuppan, Podiwee1, and Podiwee2) using a 7‐bp deletion marker at vg1126954336 in LOC_Os11g44580. LD 24 was used as the resistant check whereas Azucena (AZ) and Nipponbare (NP) was used as the susceptible checks. Lane 1–100‐bp ladder Lane 2—LD 24 Lane 3—Heendikwee1 Lane 4—Podiwee1 Lane 5—Heendikwee2 Lane 6—Moddai Karuppan Lane 7—Heendikwee3 Lane 8—Podiwee2 Lane 9—Azucena Lane 10—Nipponbare Lane 11—(−) control Lane 12–100‐bp ladder Note—Accessions available at the University of Aberdeen included three distinct Heendikwees, two Podiwees (Podiwee1 and Podiwee2), and one Moddai Karuppan. Corresponding IRGC numbers are provided in Table S3 and accessions with similar names were distinguished using numerical identifiers (e.g., Heendikwee 1, 2, and 3) as described in Munasinghe and Price, 2016. To resolve ambiguity in accession identity, International Rice gene bank codes (IRGC) were cross‐referenced with 3K Rice Genome Project IRIS identifiers as curated in RiceVarMap (2). None of the six accessions matched directly with the RiceVarMap (2) entries containing the polymorphisms of interest. However, IRIS_313‐9831 (PODIWEE) corresponds to a purified line of Podiwee1(IRGC 11938), as reported in Munasinghe and Price (2016), while IRIS_313‐9862 (MODDAI_KARUPPAN) in RiceVarMap2 is a purified derived of Moddai Karuppan (IRGC 126139) described in the same study. Despite uncertainties in accession correspondence across databases, all accessions were included in phenotypic screening for resistance, under the assumption that similarly named cultivars are likely related and may share candidate diagnostic polymorphisms.

**Table S1:** Genes in the targeted region annotated as Resistant genes.
**Table S2:** Strict criteria by which the annotated resistant genes in the targeted region was screened through Bioinformatic analysis. Initially, each gene is assessed for its presence in both KPM and LD24 (inspecting BAM alignment in IGV). Following this, the genes are examined for shared alleles between LD24 and KPM that are distinct from those in VN and NP. The rarity of the allele is determined through an analysis of RiceVarMap. The selection criteria must be applied sequentially from left to right. If a gene does not meet a specific criterion, no further criteria are evaluated, and the subsequent fields are left blank.
**Table S3:** Results of nematode screening and molecular screening experiments (with complete information on the drought scores). In phenotyping only accessions having gall counts for four or more replicates were used for further analysis.
**Table S4:** Results of the nematode screening experiment of individual plants of VELLAI for the 7‐bp marker, T SNP marker and the CR24 marker.

## Data Availability

The raw sequence data produced in Lahari et al. (2019) is available upon request from Adam Price.

## References

[pld370182-bib-0001] Abad, P. , B. Favery , M. Rosso , and P. Castagnone‐Sereno . 2003. “Root‐Knot Nematode Parasitism and Host Response: Molecular Basis of a Sophisticated Interaction.” Molecular Plant Pathology 4, no. 4: 217–224. 10.1046/j.1364-3703.2003.00170.x. 20569382

[pld370182-bib-0002] Amarasinghe, L. D. , and K. H. D. J. K. Hemachandra . 2020. “Meloidogyne Graminicola Infestation in Selected Sri Lankan Rice Varieties, *Oryza sativa* L. and Nemato‐Toxic Effect of *Trichoderma viride* to Reduce Infectivity.” Journal of Science of the University of Kelaniya 13, no. 11: 18–34. 10.4038/josuk.v13i0.8021.

[pld370182-bib-0003] Bimpong, I. K. , A. L. Carpena , M. S. Mendioro , et al. 2010. “Evaluation of *Oryza sativa* x *O. glaberrima* Derived Progenies for Resistance to Rootknot Nematode and Identification of Introgressed Alien Chromosome Segments Using SSR Markers.” African Journal of Biotechnology 9, no. 26: 3988–3997. 10.4314/ajb.v9i26.

[pld370182-bib-0004] Bridge, J. , and S. L. J. Page . 1982. “The Rice Root‐Knot Nematode, *Meloidogyne graminicola*, on Deep Water Rice (*Oryza sativa* subsp. *indica*).” Rev Nematol 5: 225–232.

[pld370182-bib-0005] Bridge, J. , R. A. Plowright , and P. D. Peng DeLiang . 2005. “Nematode Parasites of Rice.” In Plant Parasitic Nematodes in Subtropical and Tropical Agriculture, 87–130. CABI Publishing. 10.1079/9780851997278.0087.

[pld370182-bib-0006] de Majnik, J. , F. C. Ogbonnaya , O. Moullet , and E. S. Lagudah . 2003. “The Cre1 and Cre3 Nematode Resistance Genes Are Located at Homeologous Loci in the Wheat Genome.” Molecular Plant‐Microbe Interactions 16, no. 12: 1129–1134. 10.1094/MPMI.2003.16.12.1129. 14651346

[pld370182-bib-0007] Dimkpa, S. O. N. , Z. Lahari , R. Shrestha , A. Douglas , G. Gheysen , and A. H. Price . 2016. “A Genome‐Wide Association Study of a Global Rice Panel Reveals Resistance in *Oryza sativa* to Root‐Knot Nematodes.” Journal of Experimental Botany 67, no. 4: 1191–1200. 10.1093/jxb/erv470. 26552884 PMC4753847

[pld370182-bib-0008] Fanelli, E. , A. Cotroneo , L. Carisio , et al. 2017. “Detection and Molecular Characterization of the Rice Root‐Knot Nematode *Meloidogyne graminicola* in Italy.” European Journal of Plant Pathology 149, no. 2: 467–476. 10.1007/s10658-017-1196-7.

[pld370182-bib-0009] Fernandez, L. , M. T. N. Cabasan , and D. De Waele . 2014. “Life Cycle of the Rice Root‐Knot Nematode *Meloidogyne graminicola* at Different Temperatures Under Non‐Flooded and Flooded Conditions.” Archives of Phytopathology and Plant Protection 47, no. 9: 1042–1049. 10.1080/03235408.2013.829627.

[pld370182-bib-0010] Gheysen, G. , and M. G. Mitchum . 2011. “How Nematodes Manipulate Plant Development Pathways for Infection.” Current Opinion in Plant Biology 14, no. 4: 415–421. 10.1016/J.PBI.2011.03.012. 21458361

[pld370182-bib-0011] Hada, A. , T. K. Dutta , N. Singh , B. Singh , V. Rai , and N. K. Singh . 2020. “Rao U A Genome‐Wide Association Study in Indian Wild Rice Accessions for Resistance to the Root‐Knot Nematode *Meloidogyne graminicola* .” PLoS One 15, no. 9: e0239085. 10.1371/journal.pone.0239085. 32960916 PMC7508375

[pld370182-bib-0012] Jena, M. , S. L. Mohapatra , R. S. Panda , S. K. Mohanty , H. N. Thatoi , and S. C. Sahu . 2013. “Genetic Loci Associated With Root‐Knot Nematode Resistance in Rice cv. Ramakrishna.” Oryza (Cuttack) 50, no. 2: 132–139.

[pld370182-bib-0013] Kawahara, Y. , M. de la Bastide , J. P. Hamilton , et al. 2013. “Improvement of the *Oryza sativa* Nipponbare Reference Genome Using Next Generation Sequence and Optical Map Data.” Rice 6, no. 1: 4. 10.1186/1939-8433-6-4. 24280374 PMC5395016

[pld370182-bib-0014] Kyndt, T. , S. Denil , A. Haegeman , et al. 2012. “Transcriptional Reprogramming by Root Knot and Migratory Nematode Infection in Rice.” New Phytologist 196, no. 3: 887–900. 10.1111/j.1469-8137.2012.04311.x. 22985291

[pld370182-bib-0015] Kyndt, T. , D. Fernandez , and G. Gheysen . 2014. “Plant‐Parasitic Nematode Infections in Rice: Molecular and Cellular Insights.” Annual Review of Phytopathology 52, no. 1: 135–153. 10.1146/annurev-phyto-102313-050111 24906129

[pld370182-bib-0016] Lahari, Z. , A. Ribeiro , P. Talukdar , et al. 2019. “QTL‐Seq Reveals a Major Root‐Knot Nematode Resistance Locus on Chromosome 11 in Rice (*Oryza sativa* L.).” Euphytica 215, no. 7: 117. 10.1007/s10681-019-2427-0. 31274875 PMC6570777

[pld370182-bib-0017] Lecouls, A. C. , G. Salesses , J. C. Minot , et al. 1997. “Spectrum of the Ma Genes for Resistance to *Meloidogyne* spp. in Myrobalan Plum.” Theoretical and Applied Genetics 95: 1325–1334.

[pld370182-bib-0018] Liu, M. Y. , H. D. Shao , Y. Y. Wu , et al. 2023. “Meloidogyne Graminicola Population Structure in China Suggests a South‐to‐North Expansion.” Plant Disease 107, no. 7: 2070–2080. 10.1094/PDIS-08-22-1796-RE. 36691277

[pld370182-bib-0019] Mantelin, S. , S. Bellafiore , and T. Kyndt . 2017. “ *Meloidogyne graminicola*: A Major Threat to Rice Agriculture.” Molecular Plant Pathology 18, no. 1: 3–15. 10.1111/mpp.12394. 26950515 PMC6638252

[pld370182-bib-0020] Mattos, V. S. , R. R. Leite , J. E. Cares , et al. 2019. “ *Oryza glumaepatula*, a New Source of Resistance to *Meloidogyne graminicola* and Histological Characterization of Its Defense Mechanisms.” Phytopathology 109, no. 11: 1941–1948. 10.1094/PHYTO-02-19-0044-R. 31215839

[pld370182-bib-0021] Milligan, S. B. , J. Bodeau , J. Yaghoobi , I. Kaloshian , P. Zabel , and V. M. Williamson . 1998. “The Root Knot Nematode Resistance Gene Mi From Tomato Is a Member of the Leucine Zipper, Nucleotide Binding, Leucine‐Rich Repeat Family of Plant Genes.” Plant Cell 10: 1307–1319. 9707531 10.1105/tpc.10.8.1307 PMC144378

[pld370182-bib-0022] Munasinghe, M. , and A. H. Price . 2016. “Genetic and Root Phenotype Diversity in Sri Lankan Rice Landraces May Be Related to Drought Resistance.” Rice (N Y) 9: 1–13. 10.1186/S12284-016-0092-7. 27189009 PMC5396129

[pld370182-bib-0023] Nair, K. P. 2019. Combating Global Warming: The Role of Crop Wild Relatives for Food Security. Springer.

[pld370182-bib-0024] Nguyen, H. T. , S. Vang , N. T. Phan , et al. 2023. “Identification and Characterization of a Virulent Population of *Meloidogyne graminicola* .” Australasian Plant Pathology 52, no. 5: 391–405. 10.1007/s13313-023-00926-8.

[pld370182-bib-0025] Plowright, R. A. , D. L. Coyne , P. Nash , and M. P. Jones . 1999. “Resistance to the Rice Nematodes *Heterodera sacchari*, *Meloidogyne graminicola* and *M. incognita* in *Oryza glaberrima* and *O. glaberrima* x *O. sativa* Interspecific Hybrids.” Nematology 1, no. 7–8: 745–751. 10.1163/156854199508775.

[pld370182-bib-0026] Rusinque, L. , C. Maleita , I. Abrantes , J. E. Palomares‐Rius , and M. L. Inácio . 2021. “ *Meloidogyne graminicola*—A Threat to Rice Production: Review Update on Distribution, Biology, Identification, and Management.” Biology‐Basel 10, no. 11: 1163. 10.3390/biology10111163. 34827156 PMC8614973

[pld370182-bib-0027] Shrestha, R. , F. Uzzo , M. J. Wilson , and A. H. Price . 2007. “Physiological and Genetic Mapping Study of Tolerance to Root‐Knot Nematode in Rice.” New Phytologist 176, no. 3: 665–672. 10.1111/j.1469-8137.2007.02185.x. 17822410

[pld370182-bib-0029] Soriano, I. R. S. , J. C. Prot , and D. M. Matias . 2000. “Expression of Tolerance for *Meloidogyne graminicola* in Rice Cultivars as Affected by Soil Type and Flooding.” Journal of Nematology 32, no. 3: 309–317. 19270982 PMC2620461

[pld370182-bib-0028] Soriano, I. R. , V. Schmit , D. S. Brar , J. C. Prot , and G. Reversat . 1999. “Resistance to Rice Root‐Knot Nematode *Meloidogyne graminicola* Identified in *Oryza longistaminata* and *O. glaberrima* .” Nematology 1, no. 4: 395–398. 10.1163/156854199508397.

[pld370182-bib-0030] Wang, X. , R. Cheng , D. Xu , et al. 2023. “MG1 Interacts With a Protease Inhibitor and Confers Resistance to Rice Root‐Knot Nematode.” Nature Communications 14, no. 1: 3354. 10.1038/s41467-023-39080-6. PMC10250356 37291108

[pld370182-bib-0031] Yoshida, S. , D. A. Forno , J. H. Cock , and K. A. Gomez . 1976. “Routine Procedure for Growing Rice Plants in Culture Solution.” In Laboratory Manual for Physiological Studies of Rice. IRRI, Manila.

[pld370182-bib-0032] Zhao, H. , W. Yao , Y. Ouyang , et al. 2015. “RiceVarMap: A Comprehensive Database of Rice Genomic Variations.” Nucleic Acids Research 43, no. D1: D1018–D1022. 10.1093/nar/gku894. 25274737 PMC4384008

