# Supplementary material for: Evidence That Sri Lanka Is the Origin of a Rare Nematode Resistance Gene in Rice
Source: Plant Direct. 2026 Jul 12;10(7):e70182. doi: 10.1002/pld3.70182 (PMC13357382; doi:10.1002/pld3.70182)
Supplement: Supplementary file 2 — Figure S1: Molecular screen of six Sri Lankan accessions (Heendikwee1, Heendikwee2, Heendikwee3, Moddai Karuppan, Podiwee1, and Podiwee2) using a 7‐bp deletion marker at vg1126954336 in LOC_Os11g44580. LD 24 was used as the resistant check whereas Azucena (AZ) and Nipponbare (NP) was used as the susceptible checks. Lane 1–100‐bp ladder Lane 2—LD 24 Lane 3—Heendikwee1 Lane 4—Podiwee1 Lane 5—Heendikwee2 Lane 6—Moddai Karuppan Lane 7—Heendikwee3 Lane 8—Podiwee2 Lane 9—Azucena Lane 10—Nipponbare Lane 11—(−) control Lane 12–100‐bp ladder Note—Accessions available at the University of Aberdeen included three distinct Heendikwees, two Podiwees (Podiwee1 and Podiwee2), and one Moddai Karuppan. Corresponding IRGC numbers are provided in Table S3 and accessions with similar names were distinguished using numerical identifiers (e.g., Heendikwee 1, 2, and 3) as described in Munasinghe and Price, 2016. To resolve ambiguity in accession identity, International Rice gene bank codes (IRGC) were cross‐referenced with 3K Rice Genome Project IRIS identifiers as curated in RiceVarMap (2). None of the six accessions matched directly with the RiceVarMap (2) entries containing the polymorphisms of interest. However, IRIS_313‐9831 (PODIWEE) corresponds to a purified line of Podiwee1(IRGC 11938), as reported in Munasinghe and Price (2016), while IRIS_313‐9862 (MODDAI_KARUPPAN) in RiceVarMap2 is a purified derived of Moddai Karuppan (IRGC 126139) described in the same study. Despite uncertainties in accession correspondence across databases, all accessions were included in phenotypic screening for resistance, under the assumption that similarly named cultivars are likely related and may share candidate diagnostic polymorphisms. [file PLD3-10-e70182-s002.docx]

Supplementary Figures


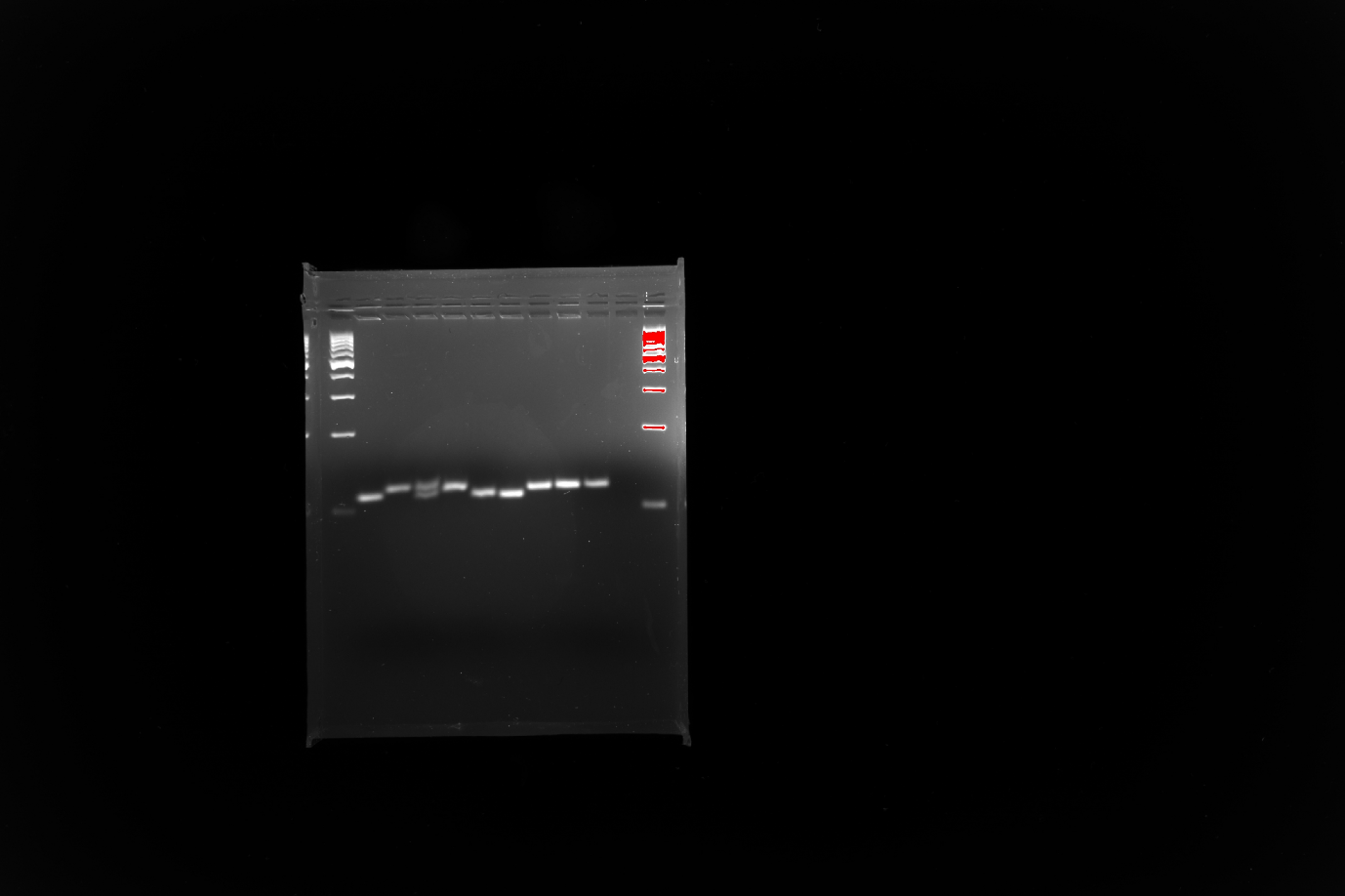


Fig. S1. Molecular screen of 6 Sri Lankan accessions (Heendikwee1, Heendikwee2, Heendikwee3, Moddai Karuppan and Podiwee1 and Podiwee2) using a 7 bp deletion marker at vg1126954336 in LOC_Os11g44580. LD 24 was used as the resistant check while Azucena (AZ) and Nipponbare (NP) was used as the susceptible checks.

Lane 1 – 100bp ladder

Lane 2 – LD 24

Lane 3 – Heendikwee1

Lane 4 - Podiwee1

Lane 5 – Heendikwee2

Lane 6 – Moddai Karuppan

Lane 7 – Heendikwee3

Lane 8 – Podiwee2

Lane 9 – Azucena

Lane 10 – Nipponbare

Lane 11 – (-) control

Lane 12 – 100 bp ladder

Note - Accessions available at the University of Aberdeen included three distinct Heendikwees, two Podiwees (Podiwee1 and Podiwee2), and one Moddai Karuppan. Corresponding IRGC numbers are provided in Table S3 and accessions with similar names were distinguished using numerical identifiers (e.g., Heendikwee 1, 2 and 3) as described in Munasinghe and Price, 2016. To resolve ambiguity in accession identity, International Rice gene bank codes (IRGC) were cross-referenced with 3K Rice Genome Project IRIS identifiers as curated in RiceVarMap (2). None of the six accessions matched directly with the RiceVarMap (2) entries containing the polymorphisms of interest. However, IRIS_313-9831 (PODIWEE) corresponds to a purified line of Podiwee1(IRGC 11938), as reported in Munasinghe and Price (2016), while IRIS_313-9862 (MODDAI_KARUPPAN) in RiceVarMap2 is a purified derived of Moddai Karuppan (IRGC 126139) described in the same study. Despite uncertainties in accession correspondence across databases, all accessions were included in phenotypic screening for resistance, under the assumption that similarly named cultivars are likely related and may share candidate diagnostic polymorphisms.

References

1. Munasinghe M, Price AH. Genetic and root phenotype diversity in Sri Lankan rice landraces may be related to drought resistance. Rice (N Y). 2016 Dec 1;9(1):1–13. doi:10.1186/S12284-016-0092-7 PubMed PMID: 27189009.

2. Zhao H, Yao W, Ouyang Y, Yang W, Wang G, Lian X, et al. RiceVarMap: a comprehensive database of rice genomic variations. Nucleic Acids Res. 2015 Jan 28;43(D1):D1018–22. doi:10.1093/nar/gku894
