# Supplementary material for: Evidence That Sri Lanka Is the Origin of a Rare Nematode Resistance Gene in Rice
Source: Plant Direct. 2026 Jul 12;10(7):e70182. doi: 10.1002/pld3.70182 (PMC13357382; doi:10.1002/pld3.70182)
Supplement: Supplementary file 1 — Data S1: Peer review. [file PLD3-10-e70182-s001.pdf]

Plant Direct
Open Access

Evidence that Sri Lanka is the origin of a rare nematode resistance gene in rice.

Adam H. Price, Mrs. Sembakutti Yashodha Sewwandhi Dharmathilaka De Silva , Ceilan Handy, Dr. Eduard Roumen, Dr. M L A M S Munasinghe.

Decision letterRound1:

January 24, 2026

Adam H. Price

University of Aberdeen School of Biological Sciences

School of Biological Sciences

Department of Plant and Soil Sciences, St Machar Drive

Aberdeen AB24 3UU.

United Kingdom

RE: Evidence that Sri Lanka is the origin of a rare nematode resistance gene in rice.

Dear Dr. Price:

Thank you for submitting to Plant Direct. All required reviews have been returned and we have now finished our evaluation of your manuscript. In light of the reviewers' and editor's comments, further revisions are needed before the paper can be accepted for publication in Plant Direct.

Please view the editors' and reviewers' comments below and use their suggestions as a guide while you work on your revision.

When uploading the revised version of this article, please be sure to include the following:

- A word document that contains your response to the reviewers. You should respond to each reviewer comment and note the changes made to the manuscript. If you do not agree with a reviewer's comment and choose not to make a suggested revision, please explain why. Please try to provide as complete an answer as possible to each reviewer's criticisms in the "response to reviewers" section.

- A tracked changes document

- A clean version of the latest version of the manuscript

To upload your revision, please click the link below.

https://plantdirect.msubmit.net/cgi-bin/main.plex?el=A3Lr7qE4A6CFn6I1A9ftdZAeDkAGtSROzcugogU9hAZ

In order to provide as timely a service as possible, we ask that your revision is resubmitted within three months after receipt of this request. If an extension is needed, please send a request, along with a brief explanation, to the editorial office at plantdirect@wiley.com .

Thank you very much for giving us an opportunity to review your work. I look forward to receiving the next version.

Sincerely,

Jonathan Fresnedo-Ramírez

Academic Editor, Plant Direct

Gustavo Macintosh

Supervising Editor, Plant Direct

-- Editor comments:

Please, follow the recommendations of the peer-review process. In addition, state the results accordingly, our peer-reviewers have noticed that the results are being overstated. If you have functional evidence on the link of the association between the 7bp deletion and the resistance phenotype, please

provide such evidence. This will make your manuscript stronger, and will align fully with Plant Direct directives.

In addition, the current version of this manuscript is riddled with grammatical errors, which is concerning. Please, work on addressing these concerns, and have a manuscript in better shape for a new round of peer-reviews.

-- Reviewers comments:

This manuscript researches the evolutionary origin and distribution of resistance to Meloidogyne graminicola in rice landraces, tied to a rare variant in LOC_Os11g44580. The approach of combining bioinformatic haplotype mining with phenotyping of geographically structured landraces is valuable, and the dataset has potential novelty (20 newly resistant accessions, geographic clustering of resistance, insight into dominance). There are a few concerns:

- the results are overstated. The MS presents a strong association between the 7bp deletion and the resistance phenotype: resistant lines almost always carry the deletion, susceptible lines almost always lack it, and geographic clustering reinforces this relationship. HOWEVER, to claim causation there should be gene function validation (knockout), protein function evidence (does the mutation change protein structure or function?), and mechanistic link (does the gene affect nematode recognition, cell death pathways, ..etc).

-Would consider replacing phrases like "very rare" with rare, "completely linked" with linked, and "the deletion is the causal resistance gene" with the is a strong genetic marker linked to resistance.

- bioinformatic filtering is subjective and weakly justified. Your filtering criteria should be explained mathematically or through clear and REPRODUCIBLE methods.

- The claim that northern Sri Lanka is the origin of resistance is not adequately supported because sampling density outside Sri Lanka is low, only Sri Lankan lines were screened, recombination and introgression history aren't analyzed. This should be reframed more conservatively.

- If your candidate gene aligns to resistance in wheat nematode systems, why does it not overlap with MG1 annotation?

-would change "bottom of chromosome" to "end" to sound more scientific

- overall, this manuscript would benefit from a very thorough review before it is resubmitted (here or elsewhere). There are many instances where the wrong tense is used (line 49: pests, line 114: has, line 121: was...), where there are parenthesis missing from citations (line 104, line 108,...), where common names are in italics, where genes are not italicized, where an abbreviation is defined multiple times, where a word is mispelled (ex: line 63: hyperplasy).

DecisionLetterRound2:

June 4, 2026

Adam H. Price

University of Aberdeen School of Biological Sciences

School of Biological Sciences

Department of Plant and Soil Sciences, St Machar Drive

Aberdeen AB24 3UU.

United Kingdom

RE: Evidence that Sri Lanka is the origin of a rare nematode resistance gene in rice.

Dear Adam:

Thank you very much for your excellent revision. Please note a very few minor points to check below. Can you attend to these and upload a final version at your earliest convenience so that we can get this to publication?

Please be sure to include the following:

-A word document that contains your response to the reviewer (very brief is fine, just note any additional text added). If you do not agree with a comment please briefly explain why.

-A tracked changes version

- A clean version of the latest version of the manuscript

To upload your revision, please click the link below.

https://plantdirect.msubmit.net/cgi-bin/main.plex?el=A5Lr6qE7B1CFn1I2A9ftdZAeDkAGtSROzcugogU9hAZ

Can you get back to me in the next day or two? Partly this is because changes are very minor, partly as we are shortly closing the eJP site. If you do actually need more time (say beyond the middle of next week?) I'll just need to ask you to submit the revision to Research Exchange, which I'd rather avoid as we're so close to completion. But if other commitments make that necessary then not a problem. Thanks.

Please note that, in addition to publishing reviewer comments, the author's responses to review comments will also be published alongside the final version of the paper. If you would not like the author's responses to be published, please contact the editorial office at plantdirect@wiley.com .

Thank you again and I look forward to seeing a final version very shortly.

Best wishes,

Jonathan

Editor, Plant Direct

-- Reviewer comments:

Reviewer #1:

The authors addressed major gaps and discussed shortcomings in the revised manuscript. There were a few grammatical and content errors that need further attention:

Line 66: Mg species should be "Meloidogyne species"

Lines 60 - 74: Lifecycle is not presented in a clear way. Very repetitive and non-linear

Line 81 & 83: root-knot nematode

Line 88: has to have

Line 133 - 135: this sentence is unclear as written, also missing a parenthesis

Line 137: use abbreviation for Vialone Nano since you defined it

Line 198: Day doesn't need to be capitalized

Line 209: have already defined J2 abbreviation

Line 226: form to from

Line 298: need a comma between Mg and selected

Line 366: Heterozygous landraces generally displayed intermediate phenotypes... This is overstating results, the lowest 2 are het.

Lines 479 - 491: Highly repetitive

Lines 497 - 525: Talk about how heterozygosity was rarely detected but then hypothesize that it may confer an evolutionary advantage for the whole paragraph. Maybe add a sentence at the end of the paragraph about how you detected heterozygosity in few landraces so evolutionary advantage may not be the case.

Response letter Round 1:

Response to reviewer's comments

Line 66: Mg species should be "Meloidogyne species" - This has been addressed in the

revised manuscript, with changes visible in the tracked changes version.

Lines 60 - 74: Lifecycle is not presented in a clear way. Very repetitive and non-linear-

The paragraph has been revised to remove repetition and streamline the interpretation. The

changes are visible in the tracked changes version.

Line 81 & 83: root-knot nematode- This has been addressed in the revised manuscript, with

changes visible in the tracked changes version.

Line 88: has to have - This has been addressed in the revised manuscript, with changes visible in the tracked changes version.

Line 133 - 135: this sentence is unclear as written, also missing a parenthesis - The sentence has been revised for clarity, and the parenthetical citation to the Rice Genome Project database has been corrected in the tracked changes version.

Line 137: use abbreviation for Vialone Nano since you defined it- This has been addressed in the revised manuscript, with changes visible in the tracked changes version.

Line 198: Day doesn't need to be capitalized- This has been addressed in the revised manuscript, with changes visible in the tracked changes version.

Line 209: have already defined J2 abbreviation- This has been addressed in the revised manuscript, with changes visible in the tracked changes version.

Line 226: form to from - This has been addressed in the revised manuscript, with changes visible in the tracked changes version.

Line 298: need a comma between Mg and selected- This has been addressed in the revised manuscript, with changes visible in the tracked changes version.

Line 366: Heterozygous landraces generally displayed intermediate phenotypes... This is overstating results, the lowest 2 are het. - We agree with the reviewer's comment and have accordingly removed this statement from the revised manuscript.

Lines 479 - 491: Highly repetitive- The paragraph has been revised to remove repetition and streamline the interpretation. The changes are visible in the tracked changes version.

Lines 497 - 525: Talk about how heterozygosity was rarely detected but then hypothesize that it may confer an evolutionary advantage for the whole paragraph. Maybe add a sentence at the end of the paragraph about how you detected heterozygosity in few landraces so evolutionary advantage may not be the case.- The paragraph has been revised to improve clarity and interpretation where appropriate. All changes are visible in the tracked changes version of the manuscript.
